# Transcriptomic and Hormonal Analyses Reveal that YUC-Mediated Auxin Biogenesis Is Involved in Shoot Regeneration from Rhizome in *Cymbidium*

**DOI:** 10.3389/fpls.2017.01866

**Published:** 2017-10-27

**Authors:** Yang Liu, Hai-Liang Zhang, He-Rong Guo, Li Xie, Rui-Zhen Zeng, Xiang-Qian Zhang, Zhi-Sheng Zhang

**Affiliations:** College of Forestry and Landscape Architecture, South China Agricultural University, Guangzhou, China

**Keywords:** auxin, *Cymbidium*, differentially expressed genes (DEGs), rhizomes, shoot regeneration

## Abstract

*Cymbidium*, one of the most important orchid genera in horticulture, can be classified into epiphytic and terrestrial species. Generally, epiphytic *Cymbidium* seedlings can be easily propagated by tissue culture, but terrestrial seedlings are difficult to propagate. To date, the molecular mechanisms underlying the differences in the ease with which terrestrial and epiphytic cymbidiums can be propagated are largely unknown. Using RNA-sequencing, quantitative reverse transcription PCR and enzyme-linked immunosorbent assay, *Cymbidium* ‘Xiaofeng’ (CXF), which can be efficiently micropropagated, and terrestrial *Cymbidium sinense* ‘Qijianbaimo’ (CSQ), which has a low regeneration ability, were used to explore the molecular mechanisms underlying the micropropagation ability of *Cymbidium* species. To this end, 447 million clean short reads were generated, and 31,264 annotated unigenes were obtained from 10 cDNA libraries. A total of 1,290 differentially expressed genes (DEGs) were identified between CXF and CSQ during shoot induction. Gene ontology (GO) enrichment analysis indicated that the DEGs were significantly enriched in auxin pathway-related GO terms. Further analysis demonstrated that YUC and GH3 family genes, which play crucial roles in the regulation of auxin/IAA (indole-3-acetic acid) metabolism, acted quickly in response to shoot induction culture *in vitro* and were closely correlated with variation in shoot regeneration between CXF and CSQ. In addition, the study showed that IAA accumulated rapidly and significantly during shoot induction in CXF compared to that in CSQ; in contrast, no significant changes in other hormones were observed between CXF and CSQ. Furthermore, shoot regeneration in CXF was inhibited by a yucasin-auxin biosynthesis inhibitor, indicating that increased IAA level is required for high-frequency shoot regeneration in CXF. In conclusion, our study revealed that YUC-mediated auxin biogenesis is involved in shoot regeneration from rhizome in *Cymbidium.*

## Introduction

*Cymbidium* is one of the most important orchid genera in horticulture, providing flowers, inflorescences, and pot plants for the orchid trade and florists (Cribb and Schuiteman, 2015). There are 52 species in the genus, which are classified into terrestrial, epiphytic or lithophytic with distribution throughout South and East Asia, the Malay Archipelago and northern and eastern Australia (Du Puy et al., 2007; Zhang et al., 2015). In China, cymbidiums are the most popular and economically important ornamental plants, among which terrestrial cymbidiums, including *Cymbidium sinense, C. ensifolium, C. faberi, C. kanran*, and *C. goeringii*, have been appreciated since the time of Confucius (551–479 BC), and remained popular in eastern Asian because of their graceful leaves, erect inflorescences and sweet scented flower (Liu, 2006). In China, they are called Chinese *Cymbidium* and regarded as a symbolic carrier of the Chinese traditional culture. By contrast, epiphytic cymbidiums, especially in their hybrid forms, have recently become a vogue of the new China’s reform and openness policies since the 1980s owing to their attractive, large, and round colorful flowers (Liu and Wang, 2005).

Micropropagation through tissue culture is the main method for producing orchid plantlets (Arditti, 2009). Previous studies have indicated that efficiencies of micropropagation vary widely among different *Cymbidium* species. Epiphytic cymbidiums and their hybrids, such as *Cymbidium* Twilight Moon ‘Day Light,’ are easy to propagate through the formation of protocorm-like bodies *in vitro* (Teixeira Da Silva and Tanaka, 2006), while terrestrial species, whose organogenesis occurs via the rhizome, are recalcitrant to propagation by tissue culture techniques (Paek and Kozai, 1998). *Cymbidium sinense*, a species native to China, has been cultured for more than a thousand years, though it is still propagated through the division of pseudobulbs *in vivo* owing to its low propagation efficiency *in vitro* (Gao et al., 2014). To improve the micropropagation efficiency, the effects of exogenous hormones, composition of culture medium, culture condition and method on the micropropagation of terrestrial species including *Cymbidium sinense* were investigated, and the results proved that it was still difficult to realize commercial production (Shimasaki and Uemoto, 1990, 1991; Zhang and Ou, 1995; Chang and Chang, 1998, 2000; Fu et al., 2000; Zhu et al., 2003; Huang and Okubo, 2005; Chiang et al., 2010; Gao et al., 2014). Hence, we proposed that genetic improvement would be an alternative approach to improving the micropropagation efficiency. However, to date, the genetic mechanisms underlying the differences in micropropagation efficiency between terrestrial and epiphytic cymbidiums remain poorly understood.

Next-generation sequencing (NGS) technologies have provided powerful tools for molecular studies of species with/without a reference genome, and the application of RNA-sequencing (RNA-seq) has enabled global transcriptome profiling in non-model plant species (Wang et al., 2009; Li et al., 2017). In recent years, NGS technologies have been applied to acquire abundant transcriptional information and drive the discovery of important genes associated with *in vitro* plant regeneration in model plants such as *Arabidopsis* (Chen et al., 2012; Xu et al., 2012; Chatfield et al., 2013), rice (Chakrabarty et al., 2010; Tie et al., 2012; Wang et al., 2014; Indoliya et al., 2016), maize (Salvo et al., 2014), and *Populus* (Bao et al., 2009) and non-model plants such as *Gracilaria lichenoides* (Wang et al., 2015) and ramie (Huang et al., 2014). However, to our knowledge, no studies have yet been conducted on transcriptome analysis in relation to the molecular mechanism of micropropagation in *Cymbidium*.

Auxins and cytokinins play key roles in shoot regeneration *in vitro*. Although significant progress has been made in revealing—at the molecular level—the auxin and cytokinin signaling and biosynthetic pathways involved in shoot regeneration in *Arabidopsis* (Duclercq et al., 2011; Motte et al., 2014; Schaller et al., 2015), the molecular pathways involved in shoot regeneration are largely unknown in *Cymbidium.* The objectives of the present study were to examine the differentially expressed genes (DEGs) between *C.* ‘Xiaofeng’ (CXF) and *C. sinense* ‘Qijianbaimo’ (CSQ) during shoot induction, to elucidate the molecular mechanism underlying the differences in micropropagation efficiency between the two types, and to provide a fundamental theoretical basis for improving the micropropagation capacity in *Cymbidium*.

## Materials and Methods

### Plant Materials

Two *Cymbidium* cultivars, CXF, and CSQ, were used in this study. CSQ is a traditional cultivar in Guangdong Province, China, and CXF is a new cultivar bred in our laboratory by combining *C.* Maureen Carter ‘Dafeng’ and *C. sinense* ‘Qijianbaimo’ (Supplementary Figure S1A).

Rhizomes of CXF and CSQ were obtained through meristem culture. Rhizomes (1 cm long) without apical meristem were cultured for 25 days on Murashige and Skoog (MS) medium (Murashige and Skoog, 1962) supplemented with 1.0 mg⋅L^-1^ 6-benzylaminopurine (6-BA), 0.5 mg⋅L^-1^ 1-naphthaleneacetic acid (NAA), 0.05% activated charcoal (w/v) and solidified with 0.7% (w/v) carrageenan. The pH of the culture medium was settled at 5.8 after carrageenan addition and before medium sterilization (121°C, 20 min). Incubation conditions were: 26 ± 1°C, PAR 13.5 μmol m^-2^s^-1^ and 12 h light per day. Newly formed rhizomes (0.5 cm long) were used for the following experiment.

### Rhizome Proliferation and Shoot Regeneration

In order to assess the multiplication efficiency, a part of the newly formed rhizomes were cultured on rhizome-proliferation medium (RPM) composed of MS basal medium, 2.0 mg⋅L^-1^ 6-BA, 1.0 mg⋅L^-1^ NAA and 0.05% activated charcoal (w/v). These rhizomes in a jar were weighed with an analytical balance in a laminar flow bench, and the initial fresh weight was individually recorded before inoculation. Another part of the rhizomes was cultured on shoot-inducing medium (SIM) which was different from RPM for NAA concentration (0.2 mg⋅L^-1^ NAA) and lacking of activated charcoal. Phytagel^TM^ (2.5 g⋅L^-1^, Sigma–Aldrich, Saint Louis, MO, United States) was used to solidify the media. In both cases, pH was set at 5.8 and cultures were kept at the same culture conditions as previously described. Eight rhizomes were inoculated in a 250 mL culture jar with plastic lid (30 mL of medium); 10 jars were used for each treatment and the experiment was repeated three times. At the end of culture (60 days), the number of shoots produced in a culture jar on SIM and the final fresh weight of rhizomes on RPM were individually recorded.

After cultured for 0, 3, 5, 10, 15, and 20 days culture period, eight rhizomes, randomly chosen from a culture jar were collected, rinsed with deionized water, and dyed in 1% acetocarmine for 4 min at room temperature. Microscope observations were carried out by using an SMZ-161BL stereo microscope (Motic^®^, Xiamen, China).

The biomass increase and frequency of shoot regeneration were calculated according to the following formulae:

Biomass increase (%) = [(final fresh weight - initial fresh weight)/initial fresh weight] × 100%

Frequency of shoot regeneration (%) = (the number of shoots formed in the culture jar/the number of rhizomes inoculated in the culture jar) × 100%

One-way ANOVA (analysis of variance) was employed to test for statistical significance using SPSS 13.0 software (SPSS Inc., Chicago, IL, United States).

### RNA Extraction, Library Construction, and Sequencing

Twenty-four newly formed rhizomes from three culture jars were separately sampled from CXF and CSQ for RNA extraction after culturing on SIM and RPM for 0, 3, 5, 10, 15, 20, and 40 days, immediately frozen in liquid nitrogen and stored at -80°C. Total RNA of each sample was extracted using a Column Plant RNAout 2.0 (Tiandz Inc., Beijing, China) according to the manufacturer’s instructions, and treated with RNase-free DNase I (Takara Bio Inc., Otsu, Japan) to remove residual DNA. RNA quality was verified using an A2100 Bioanalyzer (Agilent Technologies, Santa Clara, CA, United States) and RNase-free agarose gel electrophoresis. Mixed RNA from 3 to 5 days samples (w:w = 1:1, stage 1) and 10, 15, 20, and 40 days samples (w:w:w:w = 1:1:1:1, stage 2) was used for library construction and sequencing utilizing 125 PE (paired-end) sequences on an Illumina HiSeq^TM^ 2000 platform; 0 day samples from CXF and CSQ were used as CK. The sequencing data had been uploaded to the NCBI “Short Read Archive” (SRA^1^), with an accession number SRP073228.

### *De Novo* Assembly and Annotation

Clean reads were selected using a Perl program by removing low-quality sequences (more than 50% bases with a quality lower than 20% in one sequence), reads with more than 5% N bases (bases unknown) and reads containing adaptor sequences (Liu and Fan, 2016), and assembled *de novo* using the Trinity program to construct the transcript sequences (Grabherr et al., 2011). The longest one was used to represent a unigene (Zhang et al., 2016). All unigenes were annotated using blastx (Altschul et al., 1997) with an E-value cut-off of 1e-5, against the following four protein databases: NCBI non-redundant protein database (nr)^2^, Clusters of Orthologous Groups of proteins database (COG)^3^, Kyoto Encyclopedia of Genes and Genomes (KEGG)^4^, and Swiss-Prot^5^.

### Filtration and Function Enrichment Analysis of Differentially Expressed Genes

Clean reads of each sample were individually mapped to the reference sequences assembled in the previous section (Supplementary Table S3) by use of the SOAPaligner/soap2 tool (Li et al., 2009). Reads uniquely mapped to a unigene were employed to calculate the gene expression level using RPKMs (reads per kilobase per million mapped reads) (Liu and Fan, 2016). DEGs between CXF and CSQ were identified using edgeR software (Robinson et al., 2010) with a false discovery rate (FDR) threshold of ≤0.001 and an absolute value of log_2_ratio ≥ 1. Representative subsets were obtained using an Interactive Venn Grapher from TBtools software^6^, and GO and KEGG enrichment analyses of these subsets were performed by TBtools software using the hyper-geometric test (Chen et al., 2016). REVIGO was applied to visualize the GO enrichment results by the use of scatter plots and interactive graphs (Supek et al., 2011). Gene expression patterns of the selected unigenes were visualized as heat maps using the ‘pheatmap’ R package^7^.

### Quantitative Reverse-Transcriptase PCR

The RNA used for quantitative reverse-transcriptase PCR (qRT-PCR) validation was the same RNA as that used for RNA-seq, and the RNA used for time course expression analysis was extracted afresh from newly formed rhizomes of CXF and CSQ cultured on SIM for 0, 3, 5, 10, 15, and 20 days. The total RNA of each sample was extracted and reverse transcribed into single-stranded cDNA using a FastQuant RT Kit (TIANGEN Biotech, Beijing, China) including gDNase according to the manufacturer’s protocol. qRT-PCR was implemented on StepOnePlus^TM^ Real-Time PCR Systems (Applied Biosystems, Foster City, CA, United States) using an SYBR Green-based PCR assay comprising 10 μl 2 × iTaq^TM^ Universal SYBR^®^ Green Supermix (Bio-Rad, Hercules, CA, United States) with first-strand cDNA as the template. Ribosomal protein S3 (RPS3) mRNA (EF065683.1) from *Cymbidium* hybrid cultivar was used as an internal reference gene (Luo et al., 2014), and primers used for qRT-PCR analysis are listed in Supplementary Table S1. The relative quantitative method, 2^-ΔΔC_T_^, was used to calculate the fold change of the target genes. Three biological replicates were performed for the qRT-PCR experiment.

### Endogenous Phytohormone Content Analysis

For endogenous auxin, cytokinin, brassinosteroid (BR), and methyl jasmonate (JA-me) content analysis, 32 rhizomes about 0.5 g fresh weight of CXF and CSQ at CK and stage 1(16 rhizomes from 3 days, 16 from 5 days) on SIM were rinsed three times with deionized water, then separately ground in liquid nitrogen. Quantifications of indole-3-acetic acid (IAA), zeatin with zeatin riboside (Z + ZR), N^6^-isopentenyladenine with N^6^-isopentenyladenosine (iP + iPR), BRs, and JA-me were carried out using an enzyme-linked immunosorbent assay (ELISA) kit, according to the recommendation of the manufacturer—China Agricultural University (Zhao et al., 2006; Wang et al., 2012). Three biological replicates were performed for each phytohormone.

### Yucasin Treatment

Newly formed rhizomes from CXF and CSQ were prepared as described in the “Plant Material” section, and inoculated in petri dishes containing SIM supplemented with 200 μM yucasin (cat. 573760, Sigma–Aldrich, St. Louis, MO, United States) (Nishimura et al., 2014). Fifteen CSQ rhizomes were inoculated on one side of the petri dish, and 15 CXF rhizomes on the other side. Each treatment had three replicates with one replicate of three petri dishes; rhizomes cultured on SIM without yucasin were the control. After exposure to cool white fluorescent light (PAR 13.5 μmol m^-2^s^-1^ and 12 h light per day) at 26 ± 1°C for 60 days, the number of CXF and CSQ shoots and the number of CXF and CSQ shoots > 1.5 cm in length in a petri dish were recorded. The frequency of shoot regeneration and frequency of shoot length > 1.5 cm were calculated according to the following formula: frequency of shoot regeneration = (the number of shoots in a petri dish/the number of inoculated rhizomes in a petri dish) × 100%; frequency of shoot length > 1.5 cm = (the number of shoots > 1.5 cm in length in a petri dish/the number of shoots in a petri dish) × 100%. Statistical significance was tested by the Student’s *t*-test using SPSS 13.0 software (SPSS Inc., Chicago, IL, United States).

## Results

### Differences in Shoot Regeneration Ability between CXF and CSQ

**FIGURE 1:**
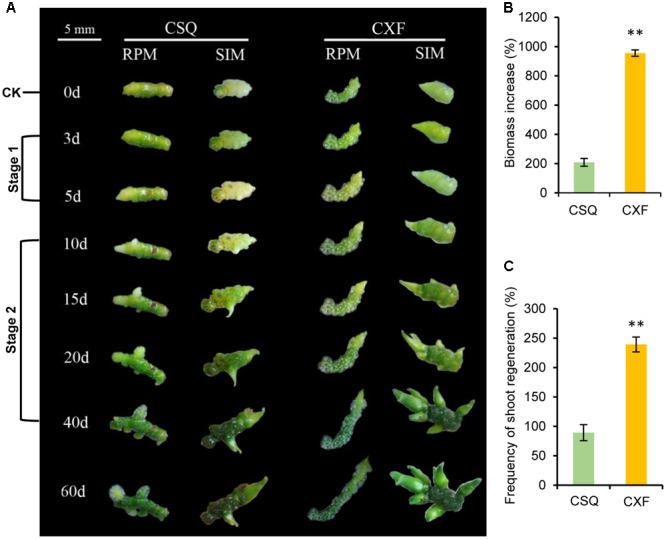
Proliferation and shoot regeneration characteristic of the rhizome from CXF and CSQ. **(A)** Morphological observation at different time points. The rhizome from CXF and CSQ was cultured on rhizome-proliferation medium (RPM) and shoot-inducing medium (SIM) for 60 days. CK, stage 1 and stage 2 were sampled for RNA-seq analysis. **(B)** Biomass increase of rhizomes cultured on RPM for 60 days compared to CK (day 0). **(C)** Frequency of shoot regeneration on SIM for 60 days. Three biological replicates (80 rhizomes per replicate) were examined. Asterisks over the bars indicate significant differences (^∗^*P* < 0.05; ^∗∗^*P* < 0.01; one-way ANOVA).

CXF exhibited a similar phenotype and the same organogenesis pathway (i.e., rhizome pathway) as CSQ (Supplementary Figure S1). Culture of rhizomes on SIM and RPM showed that the frequency of shoot regeneration in CXF was significantly higher than that in CSQ on SIM (**Figure 1C**) and shoots of CXF were obviously larger than those of CSQ (**Figure 1A**). Further observation by stereomicroscopy indicated that the apex of each CXF rhizome had formed a well-developed shoot, whereas the apex of each CSQ rhizome exhibited no shoot elongation after being cultured for 5 days (Supplementary Figure S2). In addition, the biomass increase for the CXF rhizome on RPM was significantly higher than that for the CSQ rhizome (**Figure 1B**). The findings proved that CXF and CSQ differ significantly (*P* < 0.01) in terms of rhizome proliferation and shoot regeneration.

### *De Novo* Transcriptome Assembly and Identification of Differentially Expressed Genes between CXF and CSQ

Approximately 52.07 g of total nucleotide were obtained from 10 samples of CXF and CSQ with a Q20 percentage of 96.95%. In total, 447,274,926 clean reads were assembled, and 89,412 unigenes with an N50 value of 1,081 bp generated (Supplementary Table S2). The length of the unigenes varied from 201 bp to 15,597 bp, with an average of 686.76 bp (Supplementary Figure S3). Of the 89,412 unigenes, 30,252 (33.83%) were annotated in “nr” database; 20,528 (22.96%) in Swiss-Prot; 11,179 (12.50%) in COG; and 7575 (8.74%) in KEGG (Supplementary Figure S4). As a result, 31,264 (34.97%) unigenes were annotated through sequence comparison with the nr, Swiss-Prot, COG, or KEGG database (Supplementary Figure S4).

**FIGURE 2:**
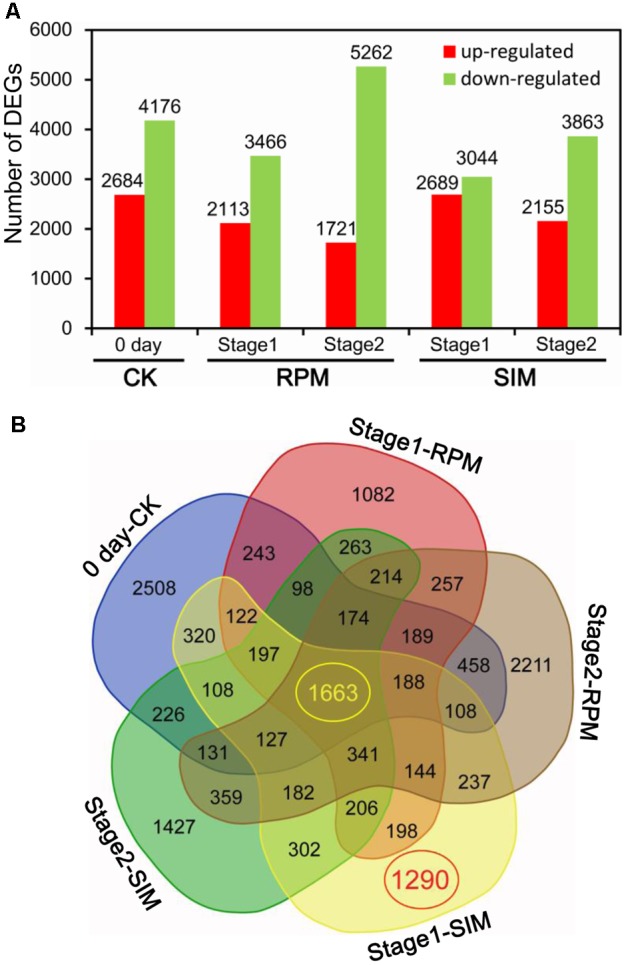
Differentially expressed genes (DEGs) between CXF and CSQ. Five pairwise comparisons were conducted between CXF and CSQ at three stages (CK or day 0, stage 1 and stage 2) on two kinds of media (SIM and RPM). **(A)** Numbers of upregulated and downregulated DEGs in each comparison. **(B)** Venn diagram demonstrating specific gene expression.

Pairwise comparisons between CXF and CSQ at three stages on both RPM and SIM identified a total of 15,573 DEGs (|log_2_ratio|≥ 1, FDR < 0.001) (**Figure 2A**). Further analysis revealed 2,508, 1,290, 1,427, 1,082, and 2,211 unique DEGs between CXF and CSQ at 0 day (CK), stage 1 on SIM, stage 2 on SIM, stage 1 on RPM, and stage 2 on RPM, respectively (**Figure 2B**). The 1,290 DEGs found at stage 1 on SIM comprised 951 upregulated genes and 399 downregulated genes, which may have been related to the difference in shoot differentiation between CXF and CSQ.

**FIGURE 3:**
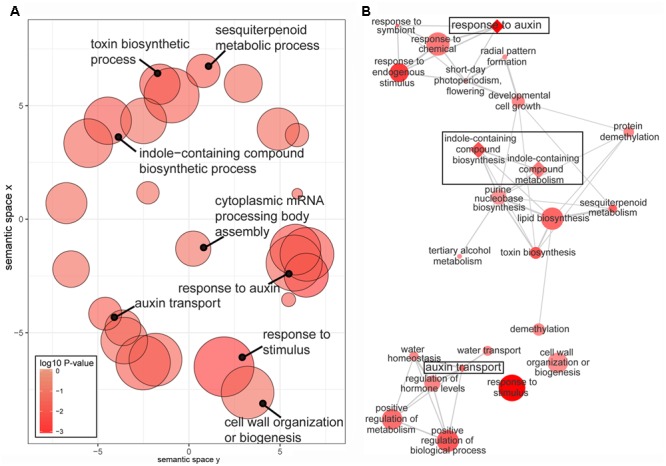
Gene ontology (GO) enrichment analysis of 1290 DEGs. **(A)** Scatter plot of enriched GO terms under the ‘biological process’ category. **(B)** Interactive graph of the enriched GO terms. The *P*-values of the GO terms were lower than 0.05. Color intensity reflects the significance of enrichment, with darker colors representing lower *P*-values. Circle radiuses (symbol size) represent frequency of the GO term in the Gene Ontology Annotation database, and bubbles of more general terms are larger. More similar nodes are positioned closer and linked by edges in the interactive graph, where the line width indicates the degree of similarity. Auxin-related GO terms are diamond shaped in the interactive graph.

The enriched GO terms associated with the 1,290 DEGs were ‘response to auxin’ and ‘auxin transport’ and ‘auxin-activated signaling pathway’ (**Figure 3A** and Supplementary Table S4). In addition, significantly overrepresented GO terms were ‘cell wall’ and ‘cellulose synthase activity,’ which may be involved in auxin-induced extension growth, including cell wall loosening and cellulose synthesis (Schopfer et al., 2002; Cosgrove, 2005). KEGG pathway analysis demonstrated that the 1,290 DEG subset was significantly enriched in a ‘plant hormone signal transduction’ pathway (Supplementary Figure S5), and eight DEGs were involved in this pathway, five of which were involved in an auxin pathway.

GO enrichment results for the 1,663 DEG subset showed that the DEGs—at all stages and on all media between CXF and CSQ were related to basic biological processes including ‘generation of precursor metabolites and energy,’ ‘proton transport,’ ‘starch metabolic process,’ and ‘electron transport chain.’ KEGG enrichment analysis also supported these findings by clusters in ‘photosynthesis,’ ‘oxidative phosphorylation,’ and ‘carbon fixation in photosynthetic organisms’ (Supplementary Figure S5). Further heat map analysis visually revealed that almost all of the DEGs (except three) that are associated with basic metabolic processes such as photosynthesis were upregulated in all five CXF samples (Supplementary Figure S6), implying that CXF may have a higher photosynthetic rate and metabolic rate than CSQ.

### Expression Patterns of Genes Related to an Auxin Pathway

**FIGURE 4:**
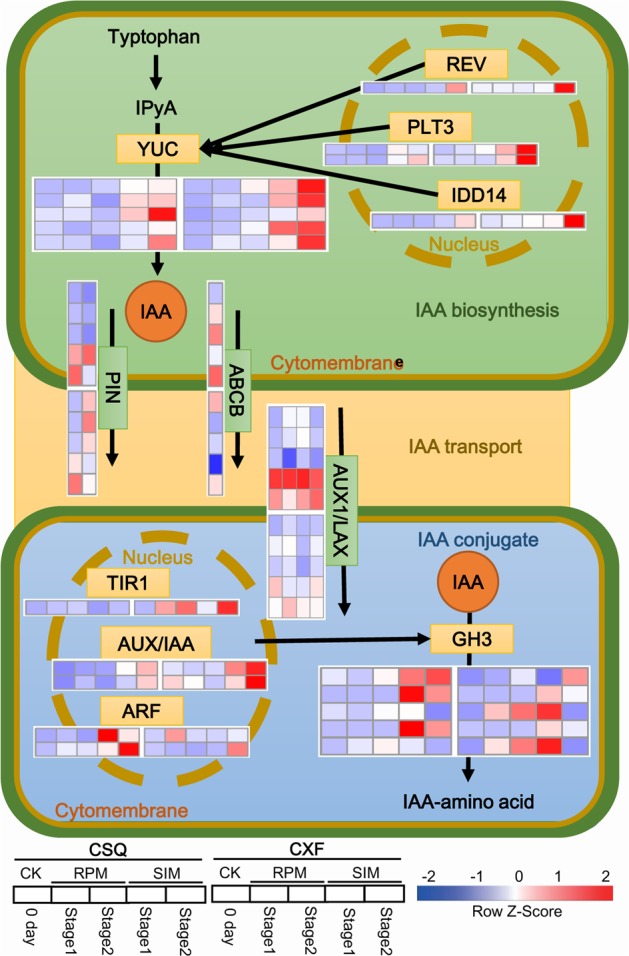
Transcriptional changes in auxin-related genes. Red indicates upregulated genes and blue indicates downregulated genes. IAA, indole-3-acetic acid; IPyA, indole-3-pyruvic acid; YUC, YUCCA, indole-3-pyruvate monooxygenase; REV, homeobox-leucine zipper protein REVOLUTA; PLT3, AP2-like ethylene-responsive transcription factor PLETHORA3; IDD14, INDETERMINATE DOMAIN14 transcription factors; ABCB, ABC transporter B family member; PIN, auxin efflux carrier family PIN; AUX1/LAX, auxin influx carrier; TIR1, transport inhibitor response 1; ARF, auxin response factor; AUX/IAA, auxin-responsive protein IAA; GH3, auxin responsive Gretchen Hagen3 gene family.

GO and KEGG enrichment results suggested that auxin-related pathways might be involved in shoot regeneration (**Figure 3** and Supplementary Table S4). Thus the expression patterns of auxin-related unigenes during shoot regeneration were further investigated, and included IAA biosynthesis genes and transport and conjugate genes, based on the DEG data. The result revealed that in the IAA biosynthesis pathway, five YUC family unigenes (unigene0001166, unigene0011312, unigene0023422, unigene0036105, unigene0036106) were upregulated on SIM, four of which had higher transcript levels in CXF than in CSQ (**Figure 4**). The expression levels of the transcription factors *REV, PLT3*, and *IDD14* (unigene0017771, unigene0027677, unigene0029375, unigene0039574) were also found to be enhanced in response to shoot induction, and CXF exhibited a higher mRNA level of these unigenes than CSQ. This finding was consistent with the observation of higher expression levels of *YUC* genes in CXF.

In the IAA conjugation pathway, five unigenes of the GH3 family were upregulated during shoot regeneration (**Figure 4**). Of these, three GH3-like unigenes (unigene0041924, unigene0050511, unigene0050513) were significantly upregulated at stage 1 and maintained a high expression level at stage 2 in CSQ, and two GH3-like unigenes (unigene0050512, unigene0050514) were markedly upregulated at stage 1, but quickly downregulated at stage 2, in CXF. In addition, TIR1 (unigene0042992), ARF (unigene0041986, unigene0057057) and AUX/IAA family unigenes (unigene0020908, unigene0030192), regulating the expression of the GH3 family gene (Ludwig-Müller, 2011), were upregulated in both CXF and CSQ during shoot regeneration. This finding was in accordance with the alteration in the expression of GH3-like unigenes. In the IAA transport pathway, four AUX1/LAX family unigenes (unigene0037234, unigene0037236, unigene0037237, unigene0037238) were upregulated during shoot regeneration (**Figure 4**), and two PIN family unigenes (unigene0031709, unigene0028984) and one member of the ABC transporters B subfamily (unigene0058835) also showed similar expression patterns with AUX1/LAX family unigenes (**Figure 4**). Detailed information concerning the expression patterns of genes related to the auxin pathways are presented in Supplementary Table S5.

To validate the RNA-seq results, 12 different genes that were found to be differentially regulated between CXF and CSQ were tested by qRT-PCR. All the genes tested displayed the same pattern of change in the qRT-PCR (Supplementary Figure S7), indicating the high level of reliability of the RNA-seq data.

### Time Course Expression Analysis of YUC-Like and GH3-Like Unigenes

**FIGURE 5:**
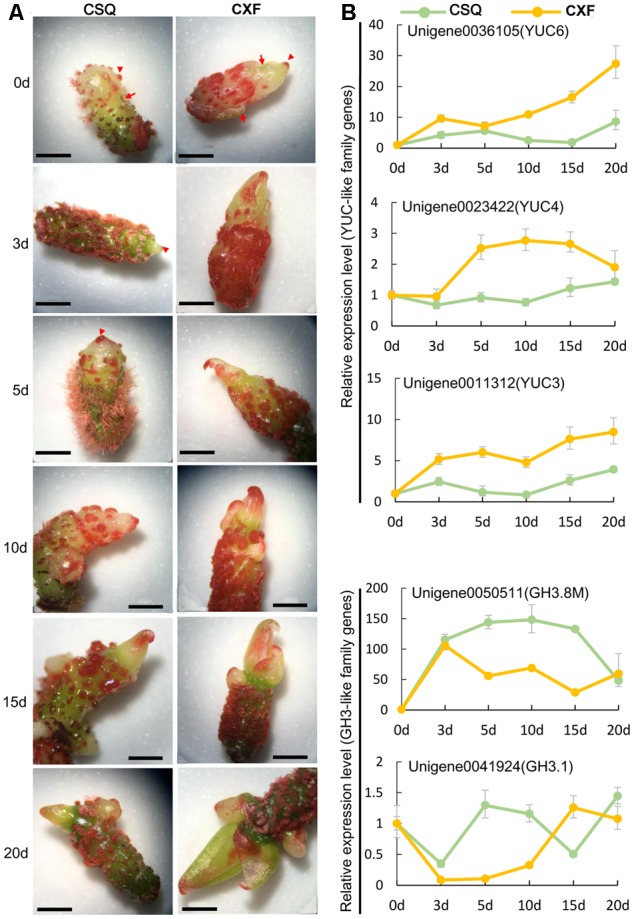
Time course of morphological structure and auxin-related gene expression during shoot regeneration of rhizomes in *Cymbidium*. **(A)** Morphological structure of CXF and CSQ cultured on SIM for 0, 3, 5, 10, 15, and 20 days. Arrowheads indicate the top of rhizomes, while arrows indicate the sheath leaves. **(B)** Time course expression analysis of YUC-like and GH3-like unigenes. The expression relative quantity at 3, 5, 10, 15, and 20 days was normalized to an expression level of 0 day. Bar = 10 mm.

The expression patterns of auxin-related genes were further analyzed using time-course gene expression data (**Figure 5**). The results showed that the YUC-like unigenes were upregulated on SIM compared with CK (0 day), and CXF had a higher transcript level of YUC-like unigenes than CSQ in all the samples tested. Interestingly, *YUC6* and *YUC3* were rapidly induced, while *YUC4* did not increase its expression level until 3 days after inoculation. The expression levels of GH3-like unigenes in CXF, such as *GH3.8M* and *GH3.1*, were lower than those of CSQ. It was noteworthy that *GH3.8M* was surprisingly upregulated over 100-fold in CSQ and maintained at a high level during shoot regeneration in culture, implying that *GH3.8M* plays a key role in IAA conjugation. These results further confirmed that the genes related to auxin pathways were significantly regulated during shoot regeneration.

### YUC-Mediated Auxin Biosynthesis Is Involved in Shoot Regeneration *in Vitro*

**FIGURE 6:**
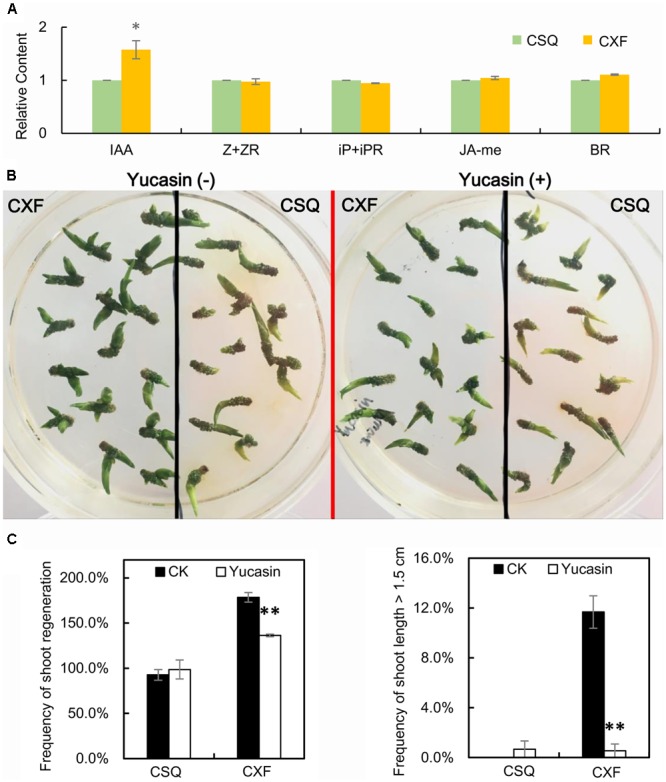
Auxin is involved in shoot regeneration of rhizomes in *Cymbidium*. **(A)** Relative content of endogenous phytohormone of rhizomes on SIM at stage 1. IAA, endogenous indole-3-acetic acid; Z + ZR, endogenous zeatin with zeatin riboside; iP + iPR, endogenous N^6^-isopentenyladenine with N^6^-isopentenyladenosine; BR, endogenous brassinosteroids; JA-me, endogenous methyl jasmonate. Three biological replicates; asterisks indicate significant differences between CXF and CSQ (^∗^*P* < 0.05; Student’s *t*-test). **(B)** Effect of yucasin on phenotypes of CXF and CSQ grown on SIM for 60 days. **(C)** Effect of yucasin on regeneration and growth of shoots of CXF and CSQ grown on SIM for 60 days. Three biological replicates, 45 rhizomes per replicate, were examined. Asterisks indicate significant differences from control (^∗^*P* < 0.05; ^∗∗^*P* < 0.01; Student’s *t*-test).

The endogenous contents of auxin (IAA), cytokinin (Z + ZR, iP + iPR), BRs and JA-me in cultured rhizomes at stage 1 on SIM were measured by ELISA. The results showed that CXF had higher levels of endogenous IAA content at stage 1 than CSQ, whereas no significant differences were found in the content of other phytohormones, including Z + ZR, iP + iPR, BR, and JA-me, between CXF and CSQ (**Figure 6A** and Supplementary Table S6). In addition, compared to CK, the relative content of endogenous IAA in CXF was significantly increased, whereas no significant alteration in endogenous IAA was observed in CSQ (Supplementary Table S6). These findings suggested that a rapid increase in endogenous auxin may have a significant effect on shoot regeneration from rhizome in *Cymbidium*.

The effect of yucasin, an auxin-biosynthesis inhibitor, on shoot regeneration was also investigated. The results showed that the shoot regeneration frequency of CXF was significantly reduced on SIM in the presence of yucasin compared with the frequency in the absence of yucasin, whereas yucasin had no effect on the shoot regeneration of CSQ (**Figures 6B,C**)—a finding that concurred with the observation of differing levels of endogenous IAA between CXF and CSQ (**Figure 6A**). Together with the link between a rapid increase in endogenous IAA and high-frequency shoot regeneration in CXF, these results suggested that YUC-mediated auxin biosynthesis may play an important role in shoot regeneration from rhizome in *Cymbidium*.

## Discussion

### Role of Auxin in Shoot Regeneration of *Cymbidium*

It is widely recognized that auxin is a plant phytohormone that plays a key role in regulating a variety of developmental processes in model plants such as *Arabidopsis* and rice, though orchids have received less attention. Nevertheless, several culture-based propagation studies have shown that certain auxin-mediated developmental responses in orchids are unique to orchid development, such as protocorm and rhizome formation (Novak et al., 2014). It has been revealed that the auxin-to-cytokinin ratio of the culture medium greatly influences the growth pattern of rhizome. High NAA-to-BA ratios in the culture medium have been found to induce rapid growth of cultured rhizomes, whereas low NAA-to-BA ratios (1:10) result in the formation of numerous branches, which in turn leads to subsequent severe shoot growth inhibition (Paek and Kozai, 1998). Exogenous auxin can not only significantly increase the fresh weight of rhizomes (Paek and Kozai, 1998), but can also promote shoot differentiation by increasing the NAA concentration (from 0 to 1 mg⋅L^-1^) (Zhu et al., 2003). These findings suggest that auxin is required for rhizome elongation and shoot regeneration. In this study, our results indicated that the frequency of shoot regeneration in CXF, a hybrid offspring of terrestrial and epiphytic cymbidiums, was significantly higher than that in CSQ, a terrestrial species native to China, and the apex of rhizomes in CXF had better developed shoots than the rhizome apexes in CSQ. Moreover, the content of endogenous IAA in CXF was significantly higher than that in CSQ during shoot induction, and the shoot regeneration ability of CXF was severely impaired by of the addition of yucasin, an auxin biosynthesis inhibitor. These results demonstrated that the difference in regeneration ability between CXF and CSQ may result from the differing IAA content in the rhizomes.

### YUC-Like and GH3-Like Unigenes Contribute to an Active IAA Level in *Cymbidium*

**FIGURE 7:**
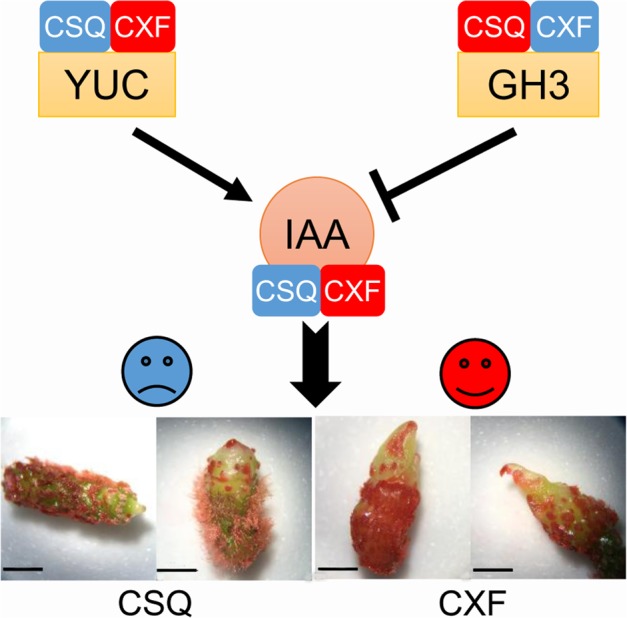
The role of YUC-mediated auxin biogenesis in shoot regeneration from rhizome in *Cymbidium*. Blue and red indicate downregulation and upregulation, respectively. YUC, YUCCA, indole-3-pyruvate monooxygenase; GH3, auxin-responsive Gretchen Hagen3 gene family; IAA, endogenous indole-3-acetic acid. Bar = 10 mm.

IAA is the most abundant endogenous auxin in plants (Simon and Petrášek, 2011). The effect of IAA on plant development mainly depends on the active IAA level, which is affected by IAA biosynthesis and conjugation processes. The Trp-dependent two-step pathway—namely, the TAA/YUC pathway—is the primary and best-defined IAA biosynthetic pathway in land plants (Yue et al., 2014). In the TAA/YUC pathway, Trp is first converted by the TAA family of aminotransferases to indole-3-pyruvate (IPA), which is then converted to IAA by the YUC family of flavin monooxygenases (Mashiguchi et al., 2011; Won et al., 2011). Given that YUC proteins catalyze a rate-limiting step of the TAA/YUC pathway (Mashiguchi et al., 2011), it has been proposed that YUC family genes play a key role in regulating IAA biosynthesis. Moreover, the endogenous IAA pool is regulated, at least partly, through negative feedback by a group of auxin-inducible *GH3* genes encoding auxin-conjugating enzymes that catalyze IAA-amino acid conjugates to reduce the level of free auxin (Ludwig-Müller, 2011). In this study, YUC-like unigenes were expressed at a high level, and GH3-like unigenes at a low level, in the rhizomes of CXF with a high regeneration capacity, compared to CSQ with a low regeneration capacity. Similar results were observed in *Arabidopsis*. For example, *YUC*-like genes *YUC1* and *YUC4* were highly expressed in *Arabidopsis* ecotype with a high regeneration capacity (Zhao et al., 2001, 2013) and the *yuc1 yuc4* double mutant reduced the shoot regeneration frequency (Cheng et al., 2013). These findings suggest that the difference in shoot regeneration ability in *Cymbidium* correlates with the differential expression of *YUC* and *GH3-*like genes, which in turn affects the active IAA content of rhizome *in vitro* (**Figure 7**).

Aside from the auxin-related pathway, GO enrichment and KEGG pathway analyses indicated that the DEGs involved in shoot regeneration were also significantly enriched in photosynthetic and metabolic pathways. In fact, several investigators have reported that the photosynthetic rate markedly influenced shoot regeneration in orchids (Norikane et al., 2010, 2013). In addition, proteomic and metabolic studies of protocorm from *Vanilla planifolia* showed that initiation of the shoot regeneration process in *Vanilla planifolia* revealed a close interrelationship between this species and both photosynthesis and glycolysis (Palama et al., 2010; Tan et al., 2013). In future studies, clarifying the relationship between photosynthesis and related metabolic processes and shoot regeneration in *Cymbidium* will aid our understanding of the molecular and physiological basis of variation in regeneration ability.

## Conclusion

CXF exhibited a high shoot regeneration ability compared to CSQ. During shoot induction, we identified 1,290 DEGs which were found to be significantly enriched in auxin-related pathways. We also observed a high expression level of YUC-like unigenes, but a low expression level of GH3-like unigenes in CXF, which led to an increase in endogenous IAA content in rhizomes during shoot regeneration, which in turn promoted shoot induction and growth.

## Author Contributions

Conceived and designed the experiments: Z-SZ, X-QZ, and YL. Performed the bioinformatics analysis: YL, H-RG, and X-QZ. Mined and analyzed the data: H-LZ, LX, Z-SZ, and YL. Performed sample preparation and culture experiments: YL, H-LZ, H-RG, and R-ZZ. Performed tissue collection, RNA isolation and qRT-PCR analysis: H-LZ, LX, and R-ZZ. Performed endogenous phytohormone content analysis: X-QZ and LX. Wrote and edited the manuscript: X-QZ, Z-SZ, and YL. All authors read and approved the final manuscript.

## Conflict of Interest Statement

The authors declare that the research was conducted in the absence of any commercial or financial relationships that could be construed as a potential conflict of interest.

## Footnotes

1 http://www.ncbi.nlm.nih.gov/sra

2 http://www.ncbi.nlm.nih.gov/

3 http://www.ncbi.nlm.nih.gov/COG/

4 http://www.kegg.jp/kegg/

5 http://www.uniprot.org/

6 https://github.com/CJ-Chen/TBtools

7 http://cran.r-project.org
