# Supplementary material for: Transcriptomic and Hormonal Analyses Reveal that YUC-Mediated Auxin Biogenesis Is Involved in Shoot Regeneration from Rhizome in Cymbidium
Source: Front Plant Sci. 2017 Oct 27;8:1866. doi: 10.3389/fpls.2017.01866 (PMC5664085; doi:10.3389/fpls.2017.01866)
Supplement: Supplementary file 1 [file Table_1.DOCX]

**TABLE S1 | Primers used for qRT-PCR.**

| Gene ID | Forward primer (5'→3') | Reverse primer (5'→3') |
| --- | --- | --- |
| Unigene0011312 | ACCTTCTCACCTATGTCAAC | ACTCCACTATCTCCTCCTC |
| Unigene0023422 | TGACTCCATTATACTCGCCACTG | TTCTCTCCCTTCCAACCTTTCG |
| Unigene0036105 | ATCGTCTGTTATTATTCGTCAC | GGAGATGGCTTTGAGTTCG |
| Unigene0057057 | CCTCCTCTGTCCTATCTGC | AACATCATTCCAAATCTCATTCC |
| Unigene0050514 | GGAGGTGGAGGAAGGGAAGG | CCAGTGACCCGCAGAATATCG |
| Unigene0050511 | GGCGGCGGAAGGAAGAGG | CGTATCTCAAGCGGTCCAATCG |
| Unigene0031709 | GCGTGATGACAAGGCTGATAC | GACCGAGGCTGAACATTGC |
| Unigene0028984 | ATGATAACGGCGGCTGAC | TGAAGTGGAAGGAGAGTAATGG |
| Unigene0019275 | CAGAATACTTCAGGTGGAGGAC | CGTTGCCGTTGCGTCTAC |
| Unigene0026607 | CCAAGCGGAATCCTGAAC | TGATGATGCCACCTAATGC |
| Unigene0054303 | CTTGGTGGCTTTCCTTTCATC | TTCTTCTTGTGTTGTGTAGTATGG |
| Unigene0042751 | AGCATCTCATTCTACAGTCTC | AAGCCAATACCAGTTCCAC |
| Unigene0037236 | GTGGTGTTGATGTTGATTC | AAGATAATGGCGAGGAAC |
| Unigene0041924 | ACTCTGCTTGGATTCTTACC | GTCACCTCTTCGTCTACTG |
| Unigene0001166 | ACGCTTCTTCATCATCTCTATC | TGACGAGGCTAGGAATGC |
| Unigene0027396 | AAAACTGGACACTGGGATGC | ATGCAACACATCCAGCGTAG |
