# Supplementary material for: Transcriptomic and Hormonal Analyses Reveal that YUC-Mediated Auxin Biogenesis Is Involved in Shoot Regeneration from Rhizome in Cymbidium
Source: Front Plant Sci. 2017 Oct 27;8:1866. doi: 10.3389/fpls.2017.01866 (PMC5664085; doi:10.3389/fpls.2017.01866)
Supplement: Supplementary file 2 [file Table_2.DOCX]

**TABLE S2 | Summary for reference transcriptome of *Cymbidium* (CXF and CSQ)**

| Before assembly | | After assembly | |
| --- | --- | --- | --- |
| Total Reads | 447274926 | **Genes Number** | 89412 |
| Total Nucleotides (nt) | 55909365750 | **N50** | 1081 |
| Q20 percentage | 96.95% | **Max length** | 15597 |
| N percentage | 0.00% | **Min length** | 201 |
| GC percentage | 48.23% | **Average length** | 686.76 |
|  |  | **GC percentage** | 40.62% |
