# Supplementary material for: Transcriptomic and Hormonal Analyses Reveal that YUC-Mediated Auxin Biogenesis Is Involved in Shoot Regeneration from Rhizome in Cymbidium
Source: Front Plant Sci. 2017 Oct 27;8:1866. doi: 10.3389/fpls.2017.01866 (PMC5664085; doi:10.3389/fpls.2017.01866)
Supplement: Supplementary file 3 [file Table_3.DOCX]

**TABLE S3 | Read quality assessment of each sample and summary of read mapping.**

| Sample | Total Raw reads | Total Clean Reads | Only Adaptor | Low quality | Mapped Reads | Unique Match |
| --- | --- | --- | --- | --- | --- | --- |
| CSQ-CK | 44653468 | 43921152 (98.36%) | 520848 (1.17%) | 209374 (0.47%) | 21621072 (49.23%) | 21601477 (49.18%) |
| CSQ-RPM-1 | 43197672 | 42424434 (98.21%) | 579220 (1.34%) | 195760 (0.45%) | 25113361 (59.20%) | 25090065 (59.14%) |
| CSQ-RPM-2 | 42384762 | 41655746 (98.28%) | 547832 (1.29%) | 179138 (0.42%) | 23849068 (57.25%) | 23825612 (57.20%) |
| CSQ-SIM-1 | 51715746 | 50841750 (98.31%) | 669452 (1.29%) | 205316 (0.40%) | 30075825 (59.16%) | 30044847 (59.09%) |
| CSQ-SIM-2 | 42558132 | 41847412 (98.33%) | 547644 (1.29%) | 165172 (0.39%) | 25273201 (60.39%) | 25248428 (60.33%) |
| CXF-CK | 48348122 | 47535874 (98.32%) | 622428 (1.29%) | 190734 (0.39%) | 25618983 (53.89%) | 25591242 (53.84%) |
| CXF-RPM-1 | 42451704 | 41683330 (98.19%) | 591178 (1.39%) | 178022 (0.42%) | 24901221 (59.74%) | 24874763 (59.68%) |
| CXF-RPM-2 | 44700600 | 43945160 (98.31%) | 545552 (1.22%) | 211692 (0.47%) | 23663356 (53.85%) | 23638020 (53.79%) |
| CXF-SIM-1 | 44751260 | 43986014 (98.29%) | 584478 (1.31%) | 182446 (0.41%) | 25553472 (58.09%) | 25527027 (58.03%) |
| CXF-SIM-2 | 50268510 | 49434054 (98.34%) | 640336 (1.27%) | 192910 (0.38%) | 28468594 (57.59%) | 28438929 (57.53%) |
