# Supplementary material for: Transcriptomic and Hormonal Analyses Reveal that YUC-Mediated Auxin Biogenesis Is Involved in Shoot Regeneration from Rhizome in Cymbidium
Source: Front Plant Sci. 2017 Oct 27;8:1866. doi: 10.3389/fpls.2017.01866 (PMC5664085; doi:10.3389/fpls.2017.01866)
Supplement: Supplementary file 6 [file Table_6.DOCX]

**TABLE S****6 | Endogenous phytohormone content of rhizomes from CXF and CSQ** **at day 0 (CK) and stage 1 (SIM-1).**

| Endogenous Phytohormone | Absolute content  (ng/g•FW) | | CSQ Increase parentage (%) | Absolute content  (ng/g•FW) | | CXF  Increase parentage  (%) |
| --- | --- | --- | --- | --- | --- | --- |
|  | **CSQ-CK** | **CSQ-SIM-1** |  | **CXF-CK** | **CXF-SIM-1** |  |
| IAA | 63.35±2.81 | 53.42±2.17 | -15.67±3.42 | 42.26±0.15** | 83.68±6.95** | 97.99±16.43** |
| Z+ZR | 7.92±0.32 | 7.59±0.26 | -4.14±3.34 | 6.91±0.51 | 7.38±0.29 | 6.81±4.17 |
| iP+iPR | 4.53±0.15 | 14.66±0.16 | 223.84±3.45 | 4.42±0.14 | 13.85±0.17 | 213.54±3.79 |
| JA-me | 22.93±0.70 | 25.36±0.58 | 10.61±2.54 | 19.36±0.69* | 26.46±0.86 | 36.66±4.45* |
| BRs | 5.20±0.17 | 4.83±0.16 | -1.62±0.68 | 4.83±0.16 | 5.35±0.23 | 10.84±4.78 |

Note: IAA: indole-3-acetic acid; Z+ZR: zeatin with zeatin riboside; iP+iPR: N^6^-isopentenyladenine with N^6^-isopentenyladenosine; JA-me: methyl jasmonate; BRs: brassinosteroids; The mean values of data were derived from three sets of biological replicates, with each replicate comprising 8 rhizomes; Three technical replicates per biological replicates; Asterisks over the bars indicate significant differences (P<0.05, *; P<0.01, **; Student’s *t*-test) between CXF and CSQ.
