# Supplementary material for: Transcriptomic and Hormonal Analyses Reveal that YUC-Mediated Auxin Biogenesis Is Involved in Shoot Regeneration from Rhizome in Cymbidium
Source: Front Plant Sci. 2017 Oct 27;8:1866. doi: 10.3389/fpls.2017.01866 (PMC5664085; doi:10.3389/fpls.2017.01866)
Supplement: Supplementary file 7 [file Image_1.PDF]

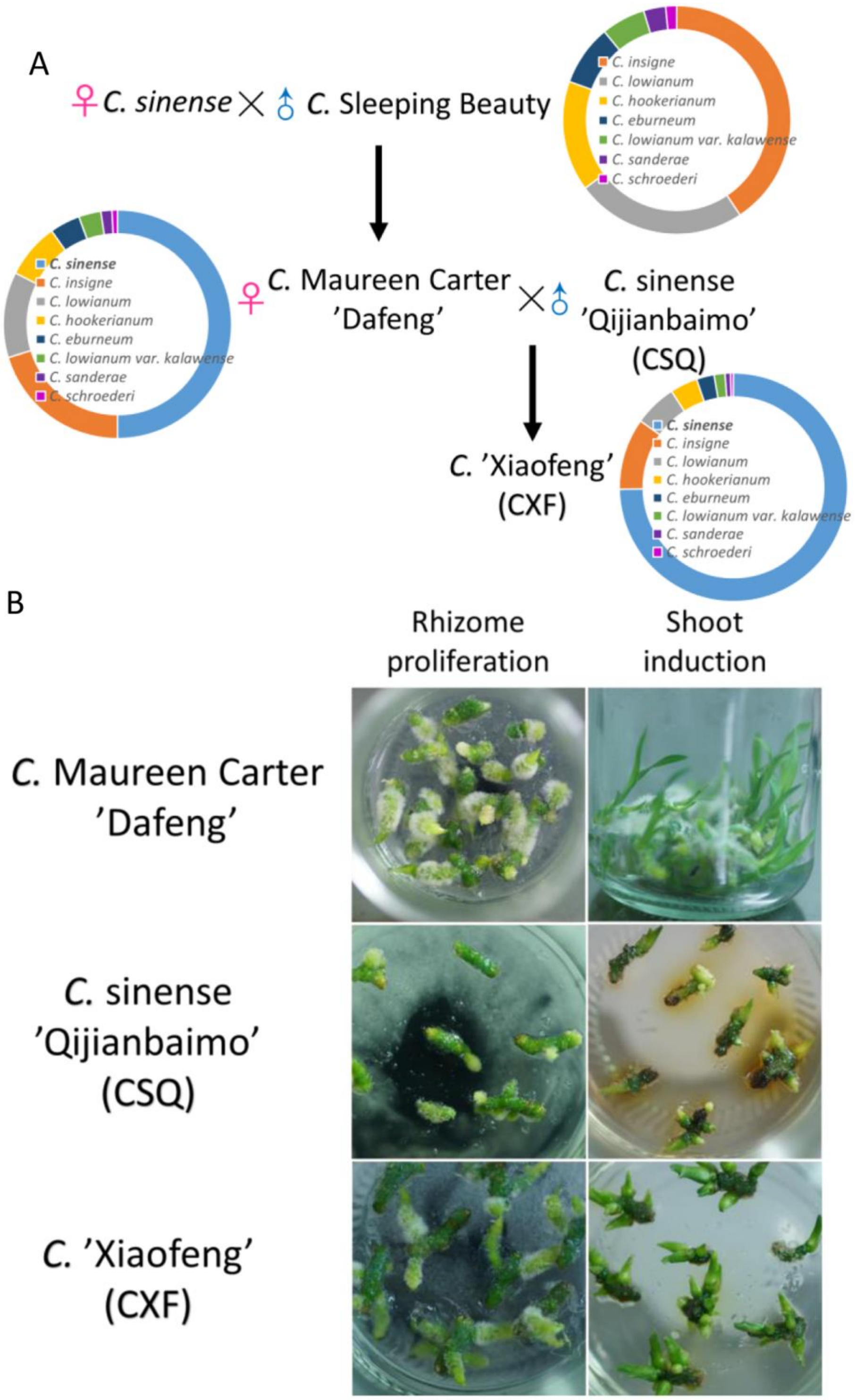

FIGURE S1 Pedigree of *Cymbidium* 'Xiaofeng' (CXF) and its characteristics in terms of suitability for micropropagation. (A) Pedigree of CXF and parentage for CXF, *C.* Maureen Carter 'Dafeng' and *C.* Sleeping Beauty. **(B)** Micropropagation characteristic of CXF, *C. sinense* 'Qijianbaimo' (CSQ) and *C.* Maureen Carter 'Dafeng' during rhizome proliferating process and shoot inducing process.
