# Supplementary material for: Transcriptomic and Hormonal Analyses Reveal that YUC-Mediated Auxin Biogenesis Is Involved in Shoot Regeneration from Rhizome in Cymbidium
Source: Front Plant Sci. 2017 Oct 27;8:1866. doi: 10.3389/fpls.2017.01866 (PMC5664085; doi:10.3389/fpls.2017.01866)
Supplement: Supplementary file 8 [file Image_2.PDF]

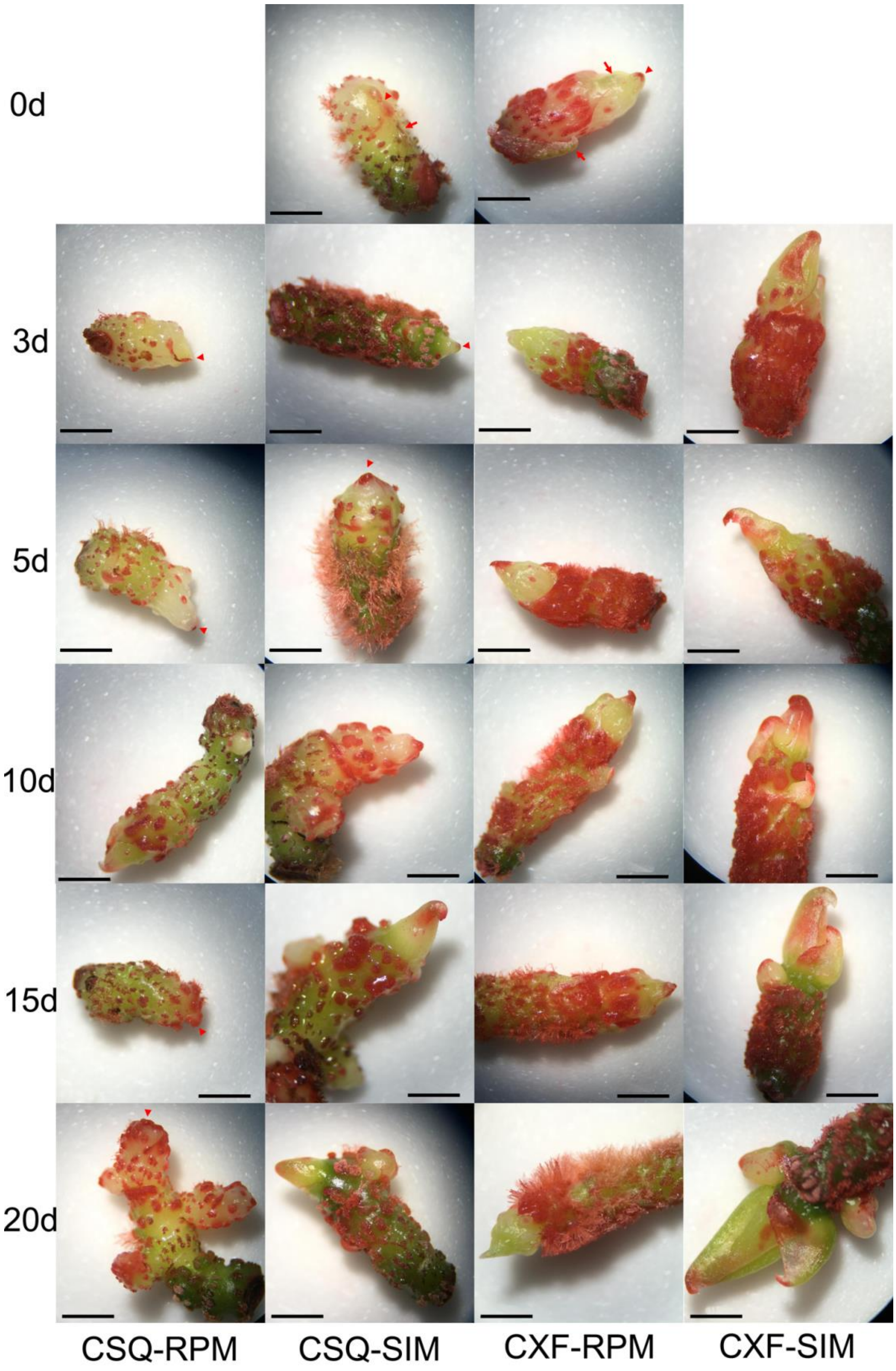

FIGURE S2 Morphological structure of rhizomes cultured on shoot-inducing medium (SIM) and rhizome-proliferation medium (RPM). Arrowheads indicate the top of rhizomes and arrows indicate the sheath leaves. Bar = 10 mm.
