# Supplementary figures and images for: Transcriptomic and Hormonal Analyses Reveal that YUC-Mediated Auxin Biogenesis Is Involved in Shoot Regeneration from Rhizome in Cymbidium
Source: Front Plant Sci. 2017 Oct 27;8:1866. doi: 10.3389/fpls.2017.01866 (PMC5664085; doi:10.3389/fpls.2017.01866)

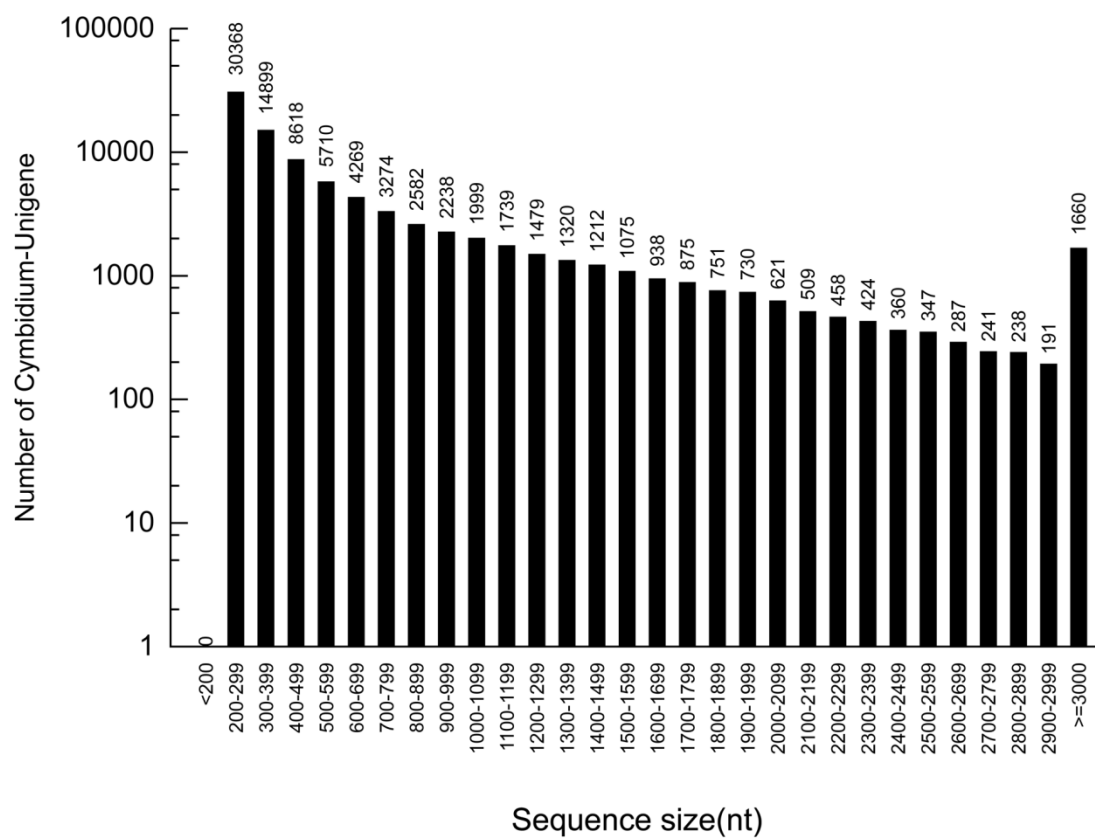

**FIGURE S3** Length distribution of assembled *Cymbidium* unigenes.

Supplement: Supplementary file 9 [file Image_3.PDF]

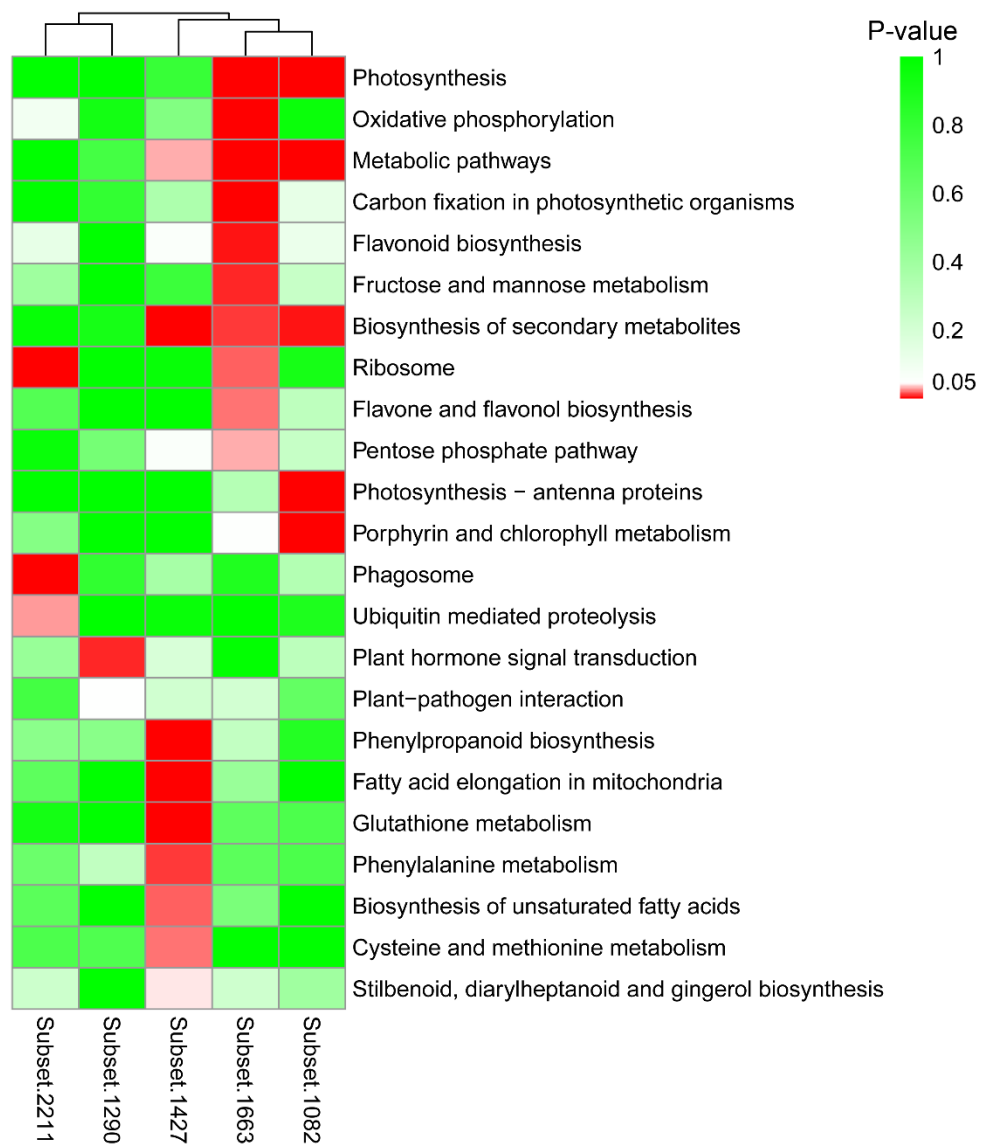

**FIGURE S5 KEGG pathway enrichment analysis of DEGs from five subsets.**

Supplement: Supplementary file 11 [file Image_5.PDF]
