# Supplementary material for: Transcriptomic and Hormonal Analyses Reveal that YUC-Mediated Auxin Biogenesis Is Involved in Shoot Regeneration from Rhizome in Cymbidium
Source: Front Plant Sci. 2017 Oct 27;8:1866. doi: 10.3389/fpls.2017.01866 (PMC5664085; doi:10.3389/fpls.2017.01866)
Supplement: Supplementary file 10 [file Image_4.PDF]

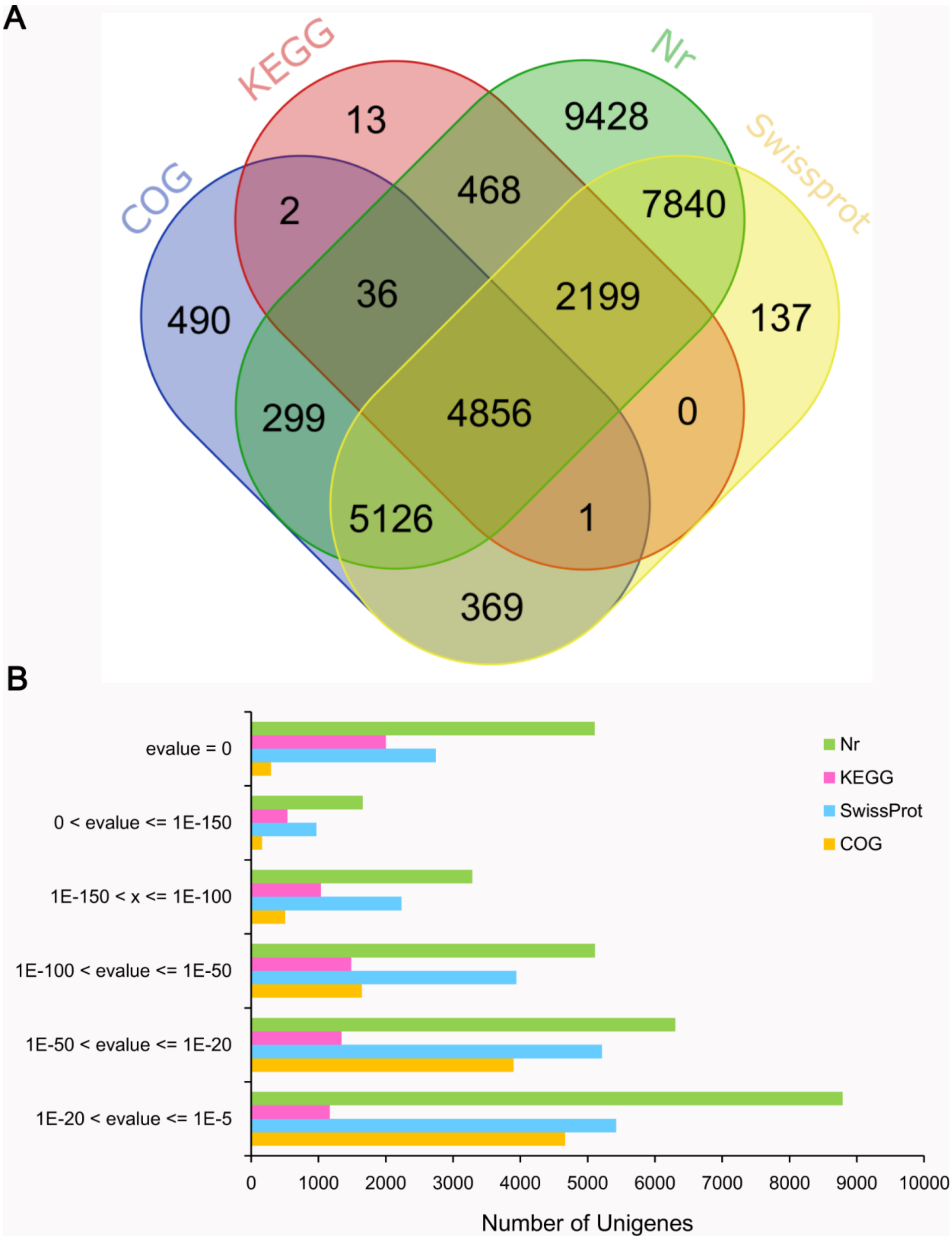

FIGURE S4 Annotation results against four protein databases. (**A**) Venn diagram of annotation results of four protein database (nr, Swiss-Prot, KEGG, and COG). The number in each color block indicated the number of unigenes that can be annotated by single or multiple databases. (**B**) Distribution of annotation E-value in four protein databases.
