# Supplementary material for: Transcriptomic and Hormonal Analyses Reveal that YUC-Mediated Auxin Biogenesis Is Involved in Shoot Regeneration from Rhizome in Cymbidium
Source: Front Plant Sci. 2017 Oct 27;8:1866. doi: 10.3389/fpls.2017.01866 (PMC5664085; doi:10.3389/fpls.2017.01866)
Supplement: Supplementary file 12 [file Image_6.PDF]

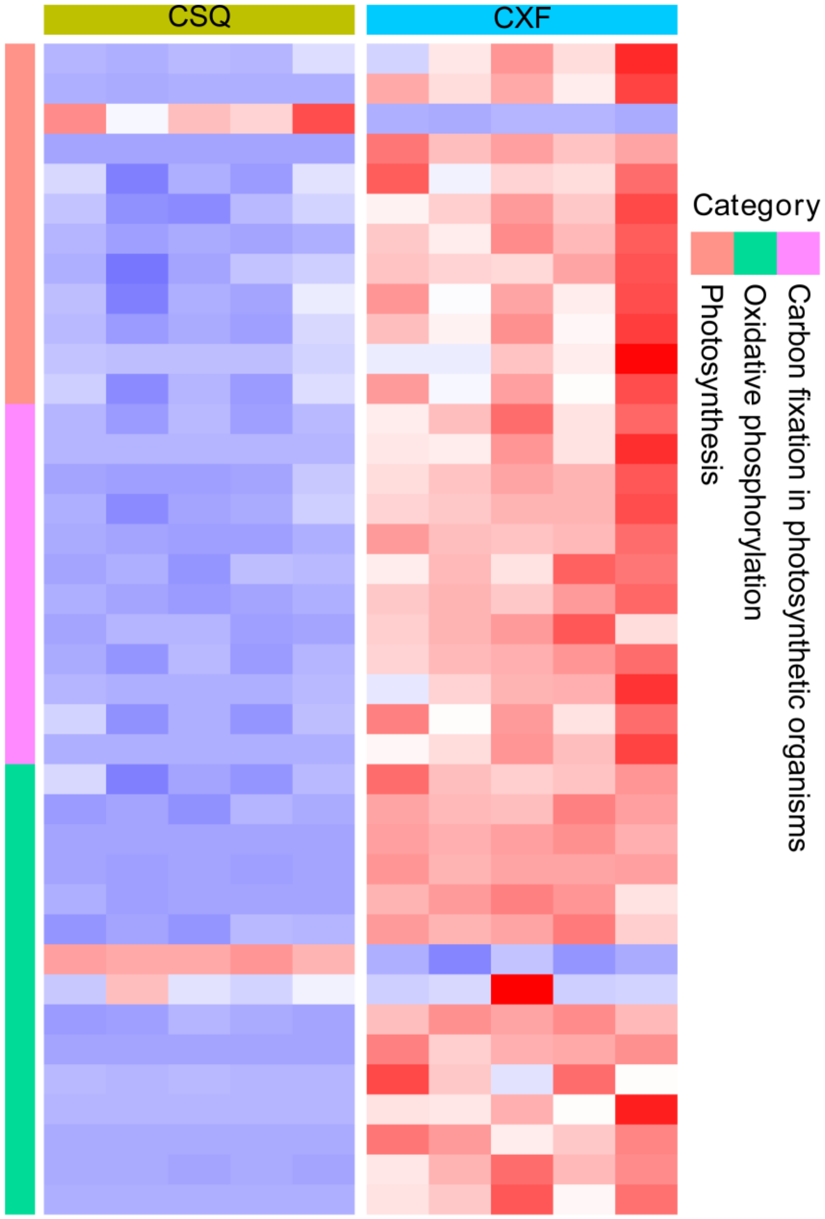

FIGURE S6 Expression patterns of enriched DEGs from subset 1663 in 10 samples of CXF and CSQ. Enriched DEGs were related to photosynthesis, carbon fixation and oxidative phosphorylation pathways.
