# Supplementary material for: Transcriptomic and Hormonal Analyses Reveal that YUC-Mediated Auxin Biogenesis Is Involved in Shoot Regeneration from Rhizome in Cymbidium
Source: Front Plant Sci. 2017 Oct 27;8:1866. doi: 10.3389/fpls.2017.01866 (PMC5664085; doi:10.3389/fpls.2017.01866)
Supplement: Supplementary file 13 [file Image_7.PDF]

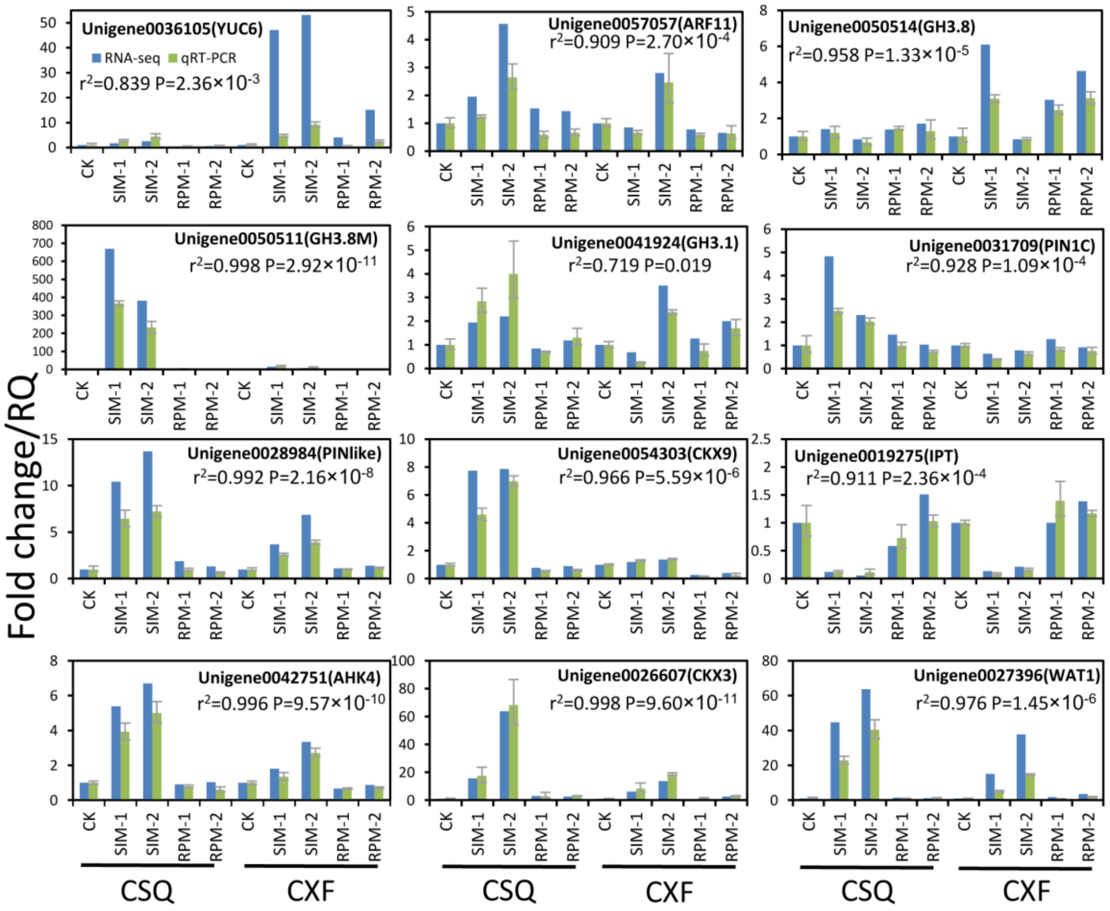

FIGURE S7 Validation of RNA-seq profiles by qRT-PCR. Fold change for RNA-seq refers to the ratios of RPKM values of SIM-1 (SIM-2, RPM-2 and RPM-2) to CK for selected transcripts, while relative quantification (RQ) value for qRT-PCR were normalized to expression level of CK. Correlation coefficient between qRT-PCR and RNA-seq were calculated and their associated P-values (P) were provided.
